# Optimisation and Efficiency Improvement of Electric Vehicles Using Computational Fluid Dynamics Modelling

**DOI:** 10.3390/e24111584

**Published:** 2022-11-01

**Authors:** Darryl Afianto, Yu Han, Peiliang Yan, Yan Yang, Anas F. A. Elbarghthi, Chuang Wen

**Affiliations:** 1 Faculty of Environment, Science and Economy, University of Exeter, Exeter EX4 4QF, UK; 2 School of Mechanical and Electrical Engineering, Suqian University, Suqian 223800, China; 3 School of Energy and Power Engineering, Beihang University, Beijing 100190, China; 4 School of Petroleum Engineering, Changzhou University, Changzhou 213164, China; 5 Faculty of Mechanical Engineering, Technical University of Liberec, Studentská 1402/2, 46117 Liberec, Czech Republic

**Keywords:** electric vehicle, electric hatchback, fuel efficiency, design, optimization, aerodynamics, computational fluid dynamics

## Abstract

Due to the rise in awareness of global warming, many attempts to increase efficiency in the automotive industry are becoming prevalent. Design optimization can be used to increase the efficiency of electric vehicles by reducing aerodynamic drag and lift. The main focus of this paper is to analyse and optimise the aerodynamic characteristics of an electric vehicle to improve efficiency of using computational fluid dynamics modelling. Multiple part modifications were used to improve the drag and lift of the electric hatchback, testing various designs and dimensions. The numerical model of the study was validated using previous experimental results obtained from the literature. Simulation results are analysed in detail, including velocity magnitude, drag coefficient, drag force and lift coefficient. The modifications achieved in this research succeeded in reducing drag and were validated through some appropriate sources. The final model has been assembled with all modifications and is represented in this research. The results show that the base model attained an aerodynamic drag coefficient of 0.464, while the final design achieved a reasonably better overall performance by recording a 10% reduction in the drag coefficient. Moreover, within individual comparison with the final model, the second model with front spitter had an insignificant improvement, limited to 1.17%, compared with 11.18% when the rear diffuser was involved separately. In addition, the lift coefficient was significantly reduced to 73%, providing better stabilities and accounting for the safety measurements, especially at high velocity. The prediction of the airflow improvement was visualised, including the pathline contours consistent with the solutions. These research results provide a considerable transformation in the transportation field and help reduce fuel expenses and global emissions.

## 1. Introduction

The sign of global warming is becoming an increasingly serious problem as a large proportion of carbon dioxide is emitted by vehicles every day [1,2,3,4,5,6]. This significant issue calls the researchers to focus on reducing vehicle emissions [7,8,9]. However, the use of fossil fuels is starting to be banned with sales of new internal combustion engine (ICE) cars banned by 2030 in the UK [10]. Currently, most hatchbacks are powered by an ICE, as such, existing vehicles will be gradually replaced by electric vehicles and consequently increase the weight from the added batteries, reinforced framework, suspensions and battery housing [11,12,13]. All these components should be addressed, since any extra weight spoils the range and efficiency of the running cars [14]. It was reported that at highway speeds, aerodynamic instabilities from drag forces are responsible for up to 50% of total fuel consumption, representing the necessity of design optimization [15].

Since the turn of the 20th century, researchers started to study the airflow around automobiles using wind tunnels, which were first introduced in 1871 and built by Wenham and Browning [16]. In 1923, the Conrad car achieved the drag force coefficient (*C_D_*) of 0.30 for automotive design, compared with 0.60 for other cars at that time and similar to road cars nowadays [17]. Due to the land and resource constraints, wind tunnels are often built for scale models of the actual cars. However, the comparison has found large amounts of the discrepancy between full-scale and scaled-down models. This can be seen in the results of the 1956 Porsche Spyder which achieved a *C_D_* value of 0.29 using a 1/5 scale model, whilst the full form obtained 0.45 *C_D_* [18]. Modern wind tunnels, such as the Volvo cars in slotted wind tunnels, can simulate 250 km/h winds with an enclosure size of 6.6 m wide and 4.1 m tall. The wind tunnel also has three configurations to simulate ground effects, scoop and both at the same time [19].

As wind tunnel tests can be very costly, the utilization of CFD software allows changes made to be tested faster and cheaper, as opposed to manufacturing and wind tunnel testing multiple prototypes with minute changes. Many studies were carried out using CFD simulations. For example, Volvo’s CFD simulations are done by creating a large rectangular box, surrounding the geometry of the car. Symmetrical wall and roof conditions are applied, with an equal velocity in the inlet and zero pressure at the outlet [19]. Another study conducted a similar simulation, the boundary conditions applied replicates real-world driving conditions with inlet speeds ranging from 70 km/h to 160 km/h [20]. A study [21] placed other boundary conditions in the inlet, such as temperature and viscosity, based on real-world locations, with a simulation for a sports car set at 300 K to simulate Croatian summer temperatures.

In 2006, research conducted on Formula Mazda race car wings found that 40% of the drag force around a car is located at the rear [22], numerous research has been done in improving the rear geometry of cars to reduce drag. A CFD research conducted in 2012 placed an arc-design diffuser under the rear bumper and extended the length of the rear whilst increasing the angle between the bumper and the ground; the design aimed to reduce drag by increasing the base pressure around the rear bumper [20]. Results found a strong correlation between increasing the diffuser length and *C_D_* reduction, with a 45 cm length, resulting in a 4.12% reduction in drag averaged across a driving speed between 70 km/h and 160 km/h in 15 km/h increments. A CFD research conducted in 2014 utilized a similar tail-plate design diffuser and found a drag reduction of 3.87% upon the use of the diffuser, with a reduction in lift coefficient of 16.62%, highlighting the effectiveness of the design [23].

The use of a diffuser can be maximized further with the application of a front lip splitter, used to decrease and guide the amount of air flowing underneath the car the splitter reduces *C_D_* and lift whilst guiding the air from the front to the diffuser in the rear [24]. The design had been implemented in 2019 research, which implemented a 35° splitter resulting in a 2.64% reduction in *C_D_* [25].

Spoilers are used as an accessory in aerodynamic adjustments placed in the rear of vehicles, used throughout motorsport, as well as production cars. Production car spoilers are placed in the trunk or roof and are designed to elongate and decrease the slope at the rear of the vehicle; this helps in delaying flow separation whilst increasing pressure in front of the spoiler and decreasing drag and lift for better stability. A 2012 CFD piece of research comparing the *C_D_* of a vehicle before and after utilizing the spoiler design found a 6.46% reduction in drag with a 17.2% reduction in the lift [24].

Further aerodynamic adjustments can be applied through the use of finlets, and vertical spoilers attached to regular spoilers designed for better flow separation at the rear and to minimize vortices. A 2019 study on a city car found that only finlets with lengths exceeding 40 mm had an impact on the aerodynamic properties of a vehicle, where the use of finlets has reduced *C_D_* by 0.76% in CFD and 1.21% in wind tunnel testing [25].

The reduction in drag coefficient can be directly correlated with the amount of fuel the car uses; with less drag, less energy is consumed when the car is cruising on a motorway maximizing fuel savings. A 1981 study researched the effect of *C_D_* reduction and fuel efficiency, with design modifications validated through a full-scale MIRA wind tunnel testing [26]. An air-bearing is placed under each wheel with adjustable tie bars connected to the truck chassis to the aerodynamic balance under the measuring platform to obtain the horizontal aerodynamic forces experienced by the vehicle. Testing was done as the vehicle travelled 365 km through the motorway at an average speed of 80 km/h and found that a 10% *C_D_* reduction had resulted in a 5.4% reduction in fuel economy. Similarly, research in 1978 highlighted the reduction in *C_D_* with fuel efficiency on a compact hatchback. The vehicle underwent full-scale wind tunnel testing at the Volkswagen climactic wind tunnel in Germany, with a similar impact area to the simulation model in this project of 2.15 m^2^. The test vehicle went through city and highway driving; results showed a 10% *C_D_* reduction had reduced the fuel consumption by 3% [27].

This paper aims to optimize and improve the efficiency of an electric hatchback using computational fluid dynamics modelling. The research will study the flow field around the vehicle body and probe the physical insight, consistent with accurate solutions. Furthermore, the effect of the different components on the flow characteristics of the vehicle is visualized, indicating the quantitative analysis of the drag coefficient, drag force and lift coefficient of the vehicle. A final enhanced model will be illustrated to facilitate future practical industrial applications and contribute to energy and emissions saving.

## 2. Numerical Domain and Simulation Method

### 2.1. Numerical Domain

**Figure 1:**
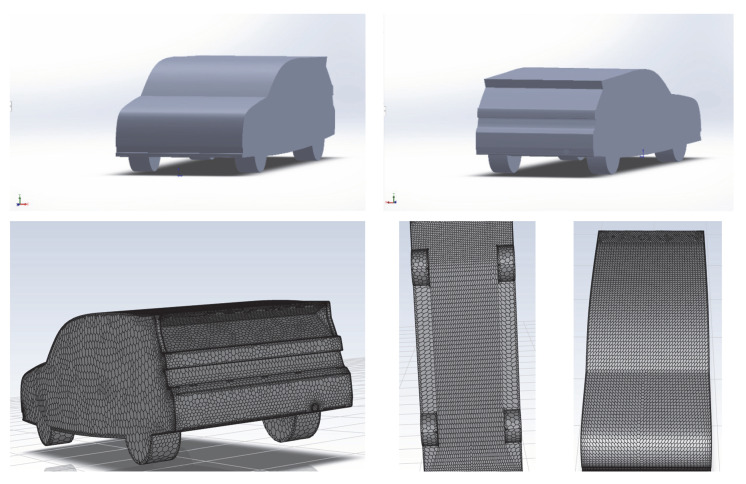
Geometry and mesh of the car for CFD modelling.

Figure 1 shows the simulation model used in this paper. The model is based on the design of the Honda Civic, with basic design elements, including bumpers, a small spoiler and splitter and wheels to simulate basic aerodynamic properties. The dimensions of the car are 4.88 m in length, 1.85 m in width and 1.5 m in height, as shown in Table 1. The model is placed in the computational domain. The dimensions of the computational domain are 7.5 m in length, 3 m in width and 1.8 m in height. Given the importance of wake characteristics above and behind the car, the length of the wind tunnel behind the car should be much longer than in front. In this study, the car model was placed 1.5 m in length from the wind tunnel inlet.

### 2.2. Governing Equations and Assumptions

The majority of production car industry calculations for velocity of flow are limited to 250 km/h, as such the flow is considered incompressible [19]. The continuity equation can be expressed as [21]:

(1) ∇u=0

Momentum equations are [21]:

(2) $$\rho \frac{Du}{Dt}=-\frac{\partial p}{\partial x}+\nabla \cdot \left(\mu \cdot \nabla u\right)+S_{Mx}$$

(3) $$\rho \frac{Dv}{Dt}=-\frac{\partial p}{\partial y}+\nabla \cdot \left(\mu \cdot \nabla v\right)+S_{My}$$

(4) $$\rho \frac{Dw}{Dt}=-\frac{\partial p}{\partial z}+\nabla \cdot \left(\mu \cdot \nabla w\right)+S_{Mz}$$

The Realizable *k*-*ε* turbulence model’s inherent stability, economy and relatively high computational accuracy make it one of the most widely used and best-known turbulence models [21].

(5) $$\frac{\partial }{\partial t}\left(\rho k\right)+\frac{\partial }{\partial x}\left(\rho ku_{j}\right)=\frac{\partial }{\partial x_{j}}\left[\left(\mu +\frac{\mu_{t}}{\sigma_{k}}\right)\frac{\partial k}{\partial x_{j}}\right]+G_{k}+G_{b}-\rho \varepsilon -Y_{M}+S_{k}$$

(6) $$\frac{\partial }{\partial t}\left(\rho \varepsilon \right)+\frac{\partial }{\partial x}\left(\rho \varepsilon u_{j}\right)=\frac{\partial }{\partial x_{j}}\left[\left(\mu +\frac{\mu_{t}}{\sigma_{\varepsilon }}\right)\frac{\partial \varepsilon }{\partial x_{j}}\right]+\rho C_{1}S\varepsilon -\rho C_{2}\frac{\varepsilon^{2}}{k+\sqrt{v\varepsilon }}+C_{1\varepsilon }\frac{\varepsilon }{k}C_{3\varepsilon }G_{b}+S_{\varepsilon }$$

The drag and lift equations are the fundamentals for the entire simulation and analysis. From the equation, it can be seen that the force a car experiences will be exponentially increased as the car accelerates. With the drag force being proportional to the drag coefficient, aerodynamic modifications towards the car in reducing *C_D_* will be critical towards reducing the force experienced by the car. Drag force and lift force are [24]:

(7) $$F_{D}=\frac{1}{2}C_{D}\rho Av^{2}$$

(8) $$F_{L}=\frac{1}{2}C_{L}\rho Av^{2}$$

### 2.3. Boundary Conditions and Simulation Methods

Boundary conditions are placed to mimic conditions in the UK, such as temperature, national speed limit, turbulence, etc. The impact area will be adjusted according to the size of the model, as such it will vary based on modifications applied. The velocity inlet is 70 km/h and the pressure is constant at 1 bar.

The coupled scheme was selected as the pressure-velocity coupling method, due to several advantages. Given the steady-state flow used in the simulation, the coupled scheme can achieve robust and efficient single-phase execution, in comparison to segregated solution methods, which solve the momentum and pressure equations separately. The coupled scheme allows the solving of momentum and pressure equations together; the fully implicit coupling is done through implicitly discretizing pressure gradient terms in the momentum equations, face mass flux, and the Rhie-Chow pressure dissipation terms.

### 2.4. Model Validation

**Figure 2:**
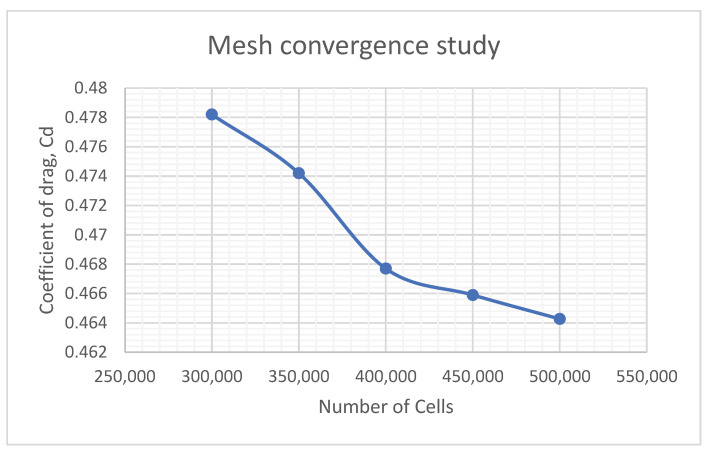
Grid independences study.

Accuracy of the simulation is essential in producing the best result. For example, the mesh convergence study on the original model is conducted to see the impact of cell count on results. It can be seen from Figure 2 that the solution increases in accuracy as more cells are applied, with a difference of 2.9% in *C_D_* from 300,000 to 500,000 cells. As the accuracy of the simulation is the priority of the CFD modelling, all models have meshed with a cell count between 500,000 and 512,000.

**Figure 3:**
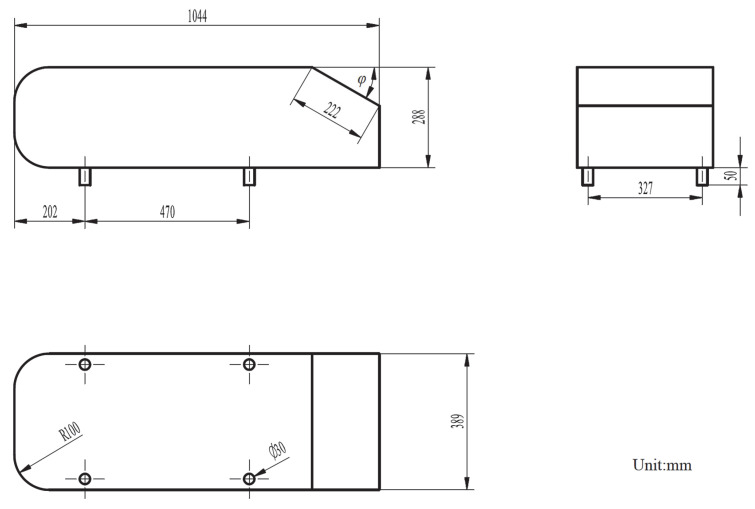
Geometry details for the model validation.

Additionally, the numerical method is validated against experimental data [28,29]. Figure 3 shows the details of the geometry used in the previous experimental measurements and we employed the rear slant angle of 20° for this validation. All the geometries and operating conditions in numerical simulations are the same with experimental studies. As can be seen, the rear area of this car model generates significant drag force due to the angular design of the rear area. In particular, the comparison of the drag force coefficient between experimental measurements and numerical results is shown in Table 2. We can see that the experimental test gave a value of 0.298 for the drag force coefficient, *C_D_*, while the numerical simulation predicts the drag force coefficient of approximately 0.311 [29]. The relative error between the numerical simulations and experiments is 4.36%. This demonstrates that our developed CFD modelling can accurately predict the flow behaviour around a vehicle and, therefore, will be used for the design and adjustments of electric vehicles.

## 3. Modification of Design

### 3.1. Base Model

**Figure 4:**
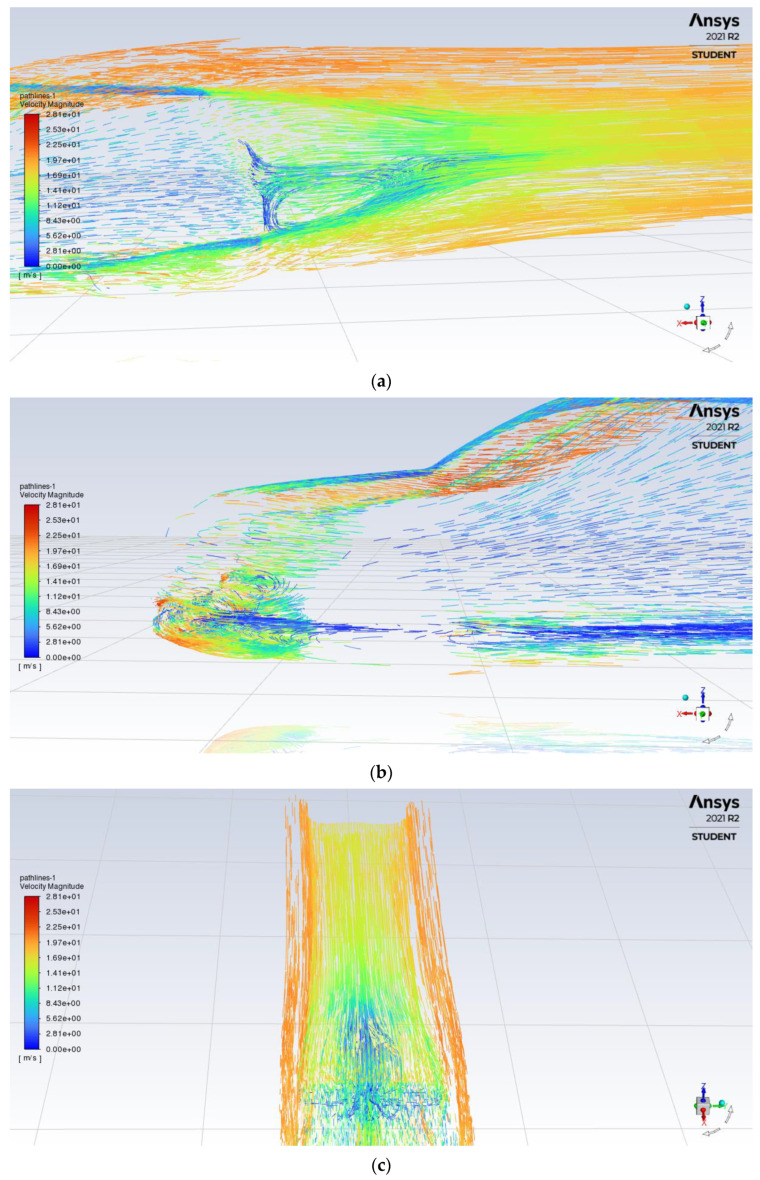
Velocity pathline of base model (**a**) rear (**b**) front (**c**) rear wake.

The original design is simulated first as a benchmark for aerodynamic improvements, analysing all aerodynamic properties of the car. From the velocity pathline results in Figure 4a, it can be seen that flow separation occurred early at the top of the car, with the large and turbulent flow coming from under the car, resulting in a large and long recirculation zone at the rear, producing higher amounts of drag and vortex creation.

The front end of the car with its sharp edge is subjected to high amounts of turbulent air, resulting in the air flowing underneath the body of the car seen in Figure 4b. Lastly, from Figure 4c we can find that there is a presence of high deflection from airflow leaving the roof and through the car sides.

### 3.2. Optimisation of Adding a Front Splitter

**Figure 5:**
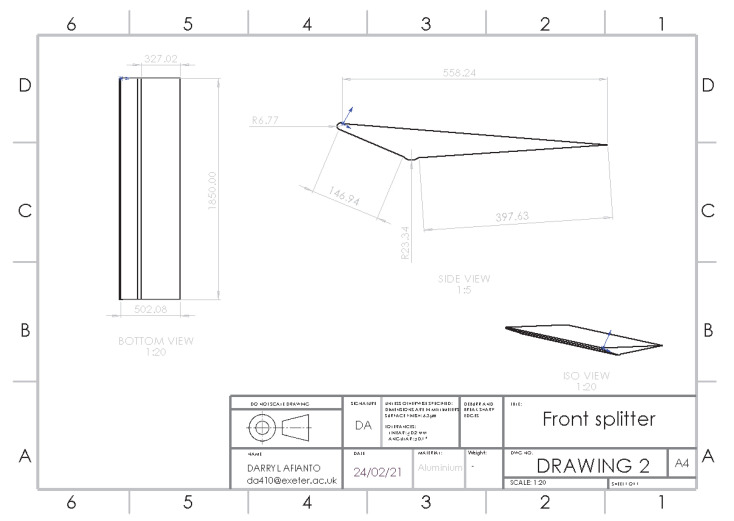
Structure of front splitter.

Tasked with improving aerodynamic properties at the front of the car, a front splitter will be designed to reduce drag and lift by redirecting the air over the car, with the small amounts of cleaner air flowing under the car guided towards the rear [24]. The splitter will utilize a rounded edge to reduce flow detachment, with the splitter reducing the angle between the front bumper and the undertray of the car based on the 35° splitter design, which saw a drag reduction of 2.64%. The splitters’ extension will slowly return in line with the body of the car to minimize turbulent flow under the car. The structure of the front splitter is shown in Figure 5.

**Figure 6:**
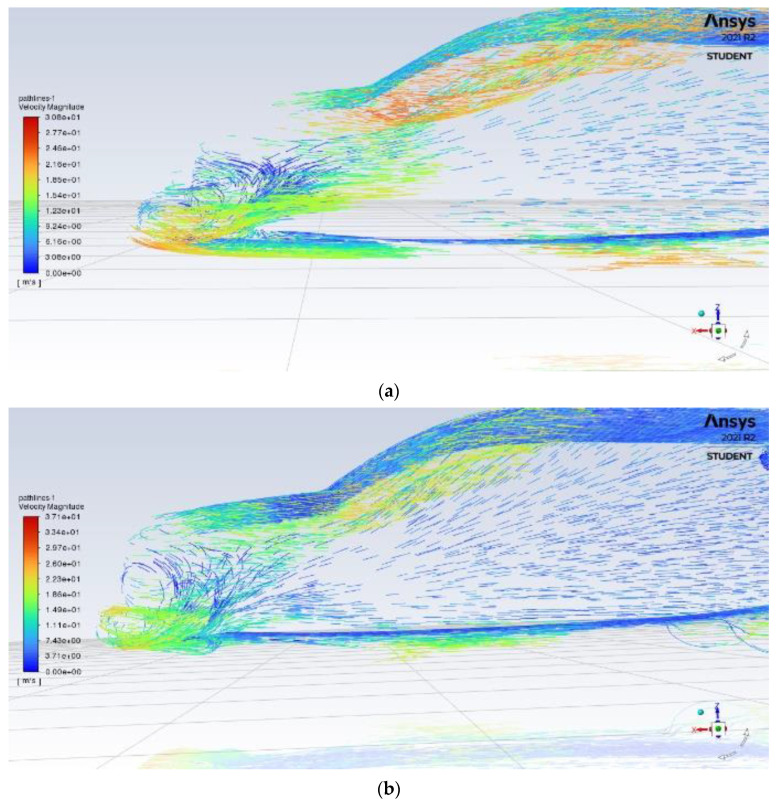
Front velocity pathline of (**a**) base model (**b**) model with front splitter.

As the air begins flowing around the car, significant turbulence can be seen underneath the front bumper in Figure 6a, with a large flow separation region causing drag, inconsistency and large distributions of velocity in the flow. The splitter’s effect can immediately be seen in Figure 6b as the flow separation region is reduced in size. In addition, the result shows that maximum turbulent kinetic energy values were reduced by 26.5%. The splitter model has a consistent flow with a smaller boundary layer and smaller velocity variance

### 3.3. Optimisation of Adding a Rear Diffuser

**Figure 7:**
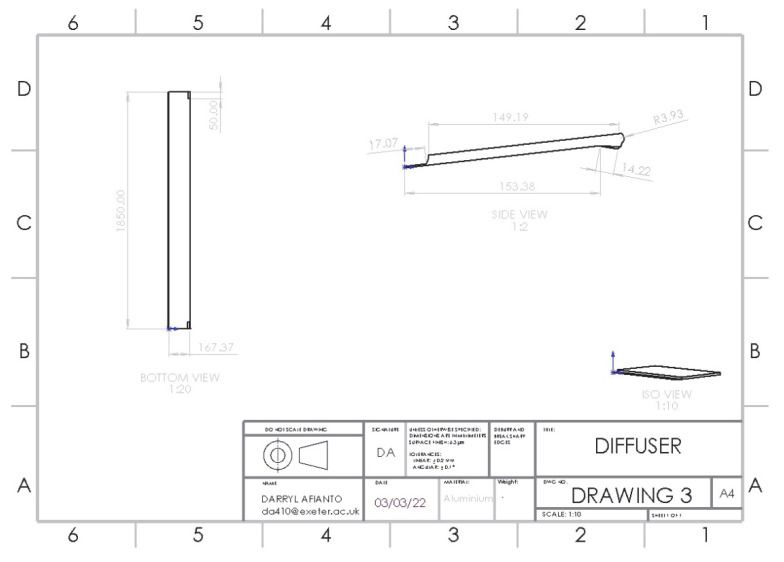
Structure of rear diffuser.

A rear diffuser can reduce drag by increasing base pressure around the rear bumper. As air flows underneath the car, the air will be lower in pressure, compared to the air at the rear of the car, and the pressure difference creates a vacuum and sucks air from the car, which increases downforce (negative lift) and reduces drag. The design is based on the arc-shaped [20] design, proven in reducing the drag of the car; research on the design also shows the correlation between the diffuser’s length and increase in *C_D_* reduction, with a length between 15 and 45 cm obtaining the best results. The design is supported further by another tail-plate design [23], which shows a reduction in *C_D_* of 3.87% and C_L_ of 16.62%. The structure of the rear diffuser is shown in Figure 7.

**Figure 8:**
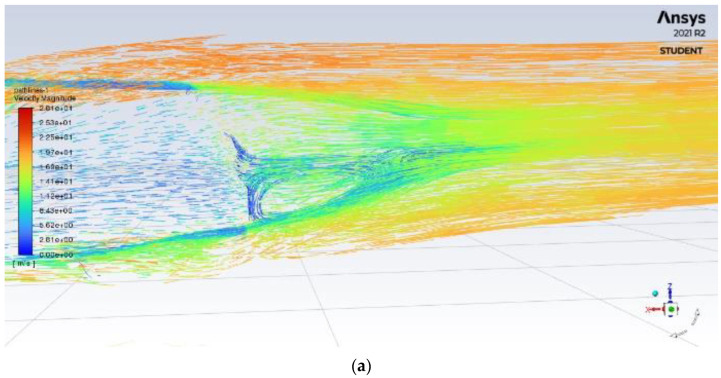

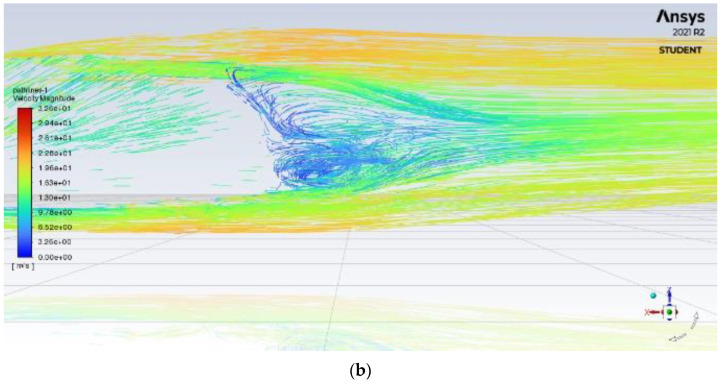
Rear velocity pathline of (**a**) base model (**b**) model with rear diffuser.

From Figure 8a, the impact of the diffuser can be seen as the air increases in velocity with a smoother flow from under the car. The diffuser utilizes Bernoulli’s principle guiding the air through the low-pressure area seen in Figure 8b, increasing the velocity; once the air pressure increases past the diffuser, the velocity reduces significantly, decreasing the lift and producing downforce. The diffuser also blocks the airflow from re-circulating in the rear, reducing the drag by decreasing the size of the re-circulation zone.

### 3.4. Optimisation of Adding a Rear Spoiler

**Figure 9:**
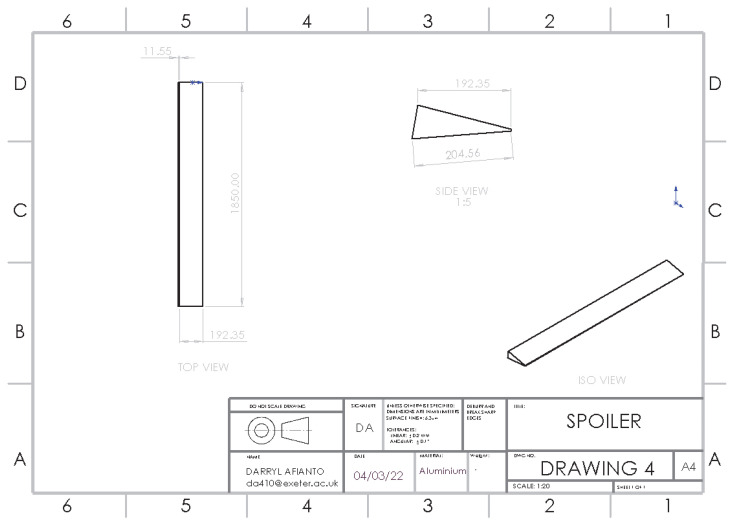
Structure of rear spoiler.

The spoiler is attached at the rear of the roof to elongate and decrease the slope at the rear of the vehicle, designed to delay flow separation and increase pressure in front of the spoiler. As the air reaches the spoiler, the high-pressure area reduces the airspeed reaching the air-circulation zone, reducing drag and lift. The spoiler design is based on a 2014 piece of research, correlating spoiler angle and reduction in the drag coefficient. The results show that the optimum spoiler angle between 10°–15° from horizontal produces the highest reduction in the lift whilst reducing drag at a good value [30]. The structure of the rear spoiler is shown in Figure 9.

**Figure 10:**
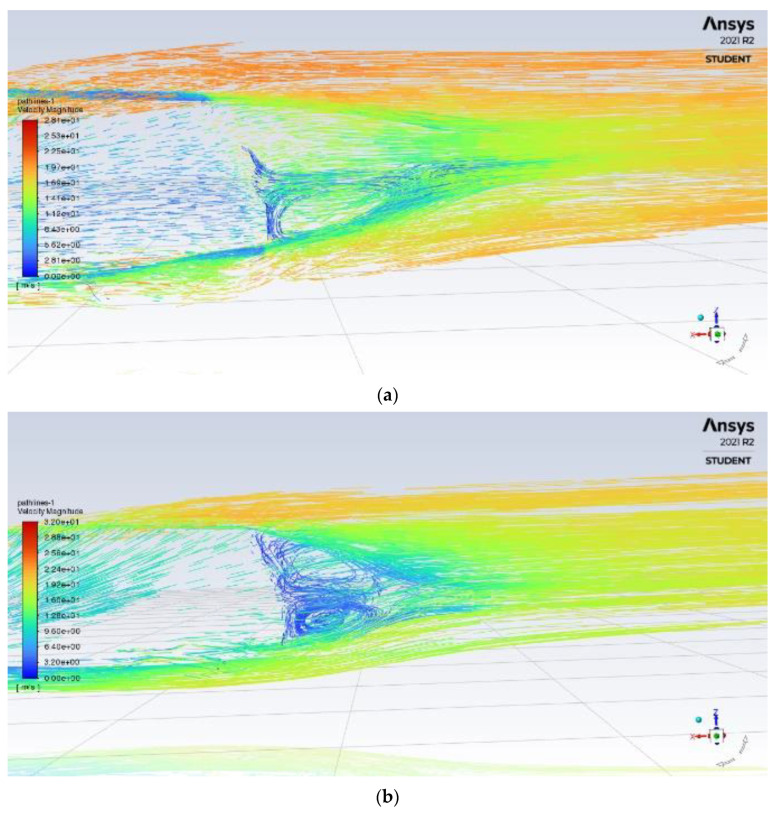
Rear velocity pathline of (**a**) base model (**b**) model with a rear spoiler.

From Figure 10, the delay in flow separation can be seen with higher velocity airflow, flowing over the circulation zone, with lower velocity air pushed towards the rear of the car. As a result, the size of recirculation is reduced with a reduction in drag seen in Table 3; similarly, turbulence is decreased as the size and flow of the wake over the car is improved.

### 3.5. Optimisation of Adding Rear Fins

**Figure 11:**
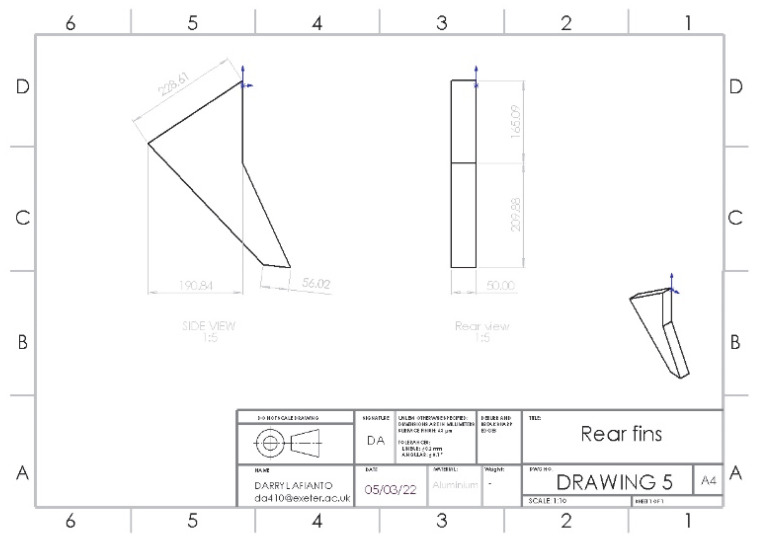
Structure of rear fins.

The rear fins are attached to the spoiler vertically, designed to reduce vortices from the side of the car and avoid deflection. The design of the fin is based on a study on fin application towards drag reduction, with 40 mm of length necessary for effective fin application. The structure of the rear fins is shown in Figure 11.

**Figure 12:**
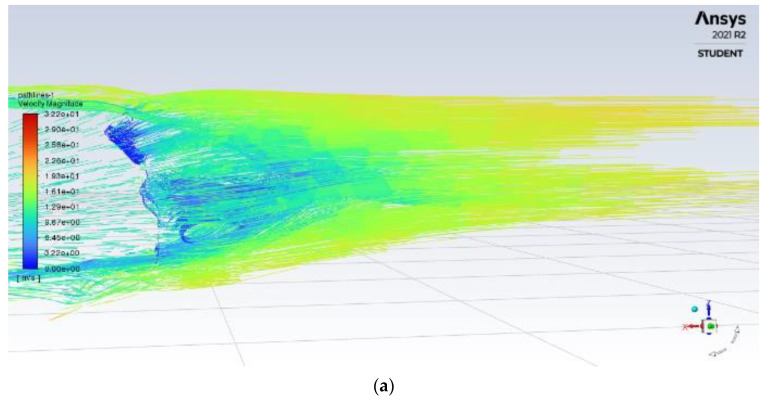

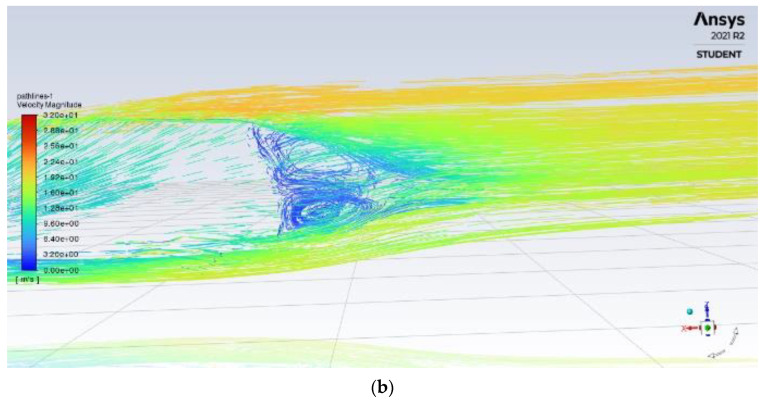
Rear velocity pathline of (**a**) base model (**b**) model with rear fins.

As the air reaches the rear of the car as shown in Figure 12, the rear fins allow for reducing the drag. Therefore, the size of the upper recirculation zone is minimized and improved with reduced turbulence values. Once the wake has formed, the flow separation between high airspeed flow and low airspeed flow can be seen, with turbulence and the size of the wake improved over the original model. Although turbulent intensity values in the circulation zone are higher, it is smaller in size and much more contained, and the turbulence behind the car has been improved significantly in the upper region with more dense and uniform flow.

### 3.6. Optimised Model

**Figure 13:**
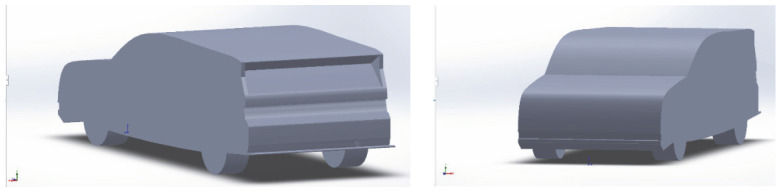
The adjusted model of the car.

**Figure 14:**
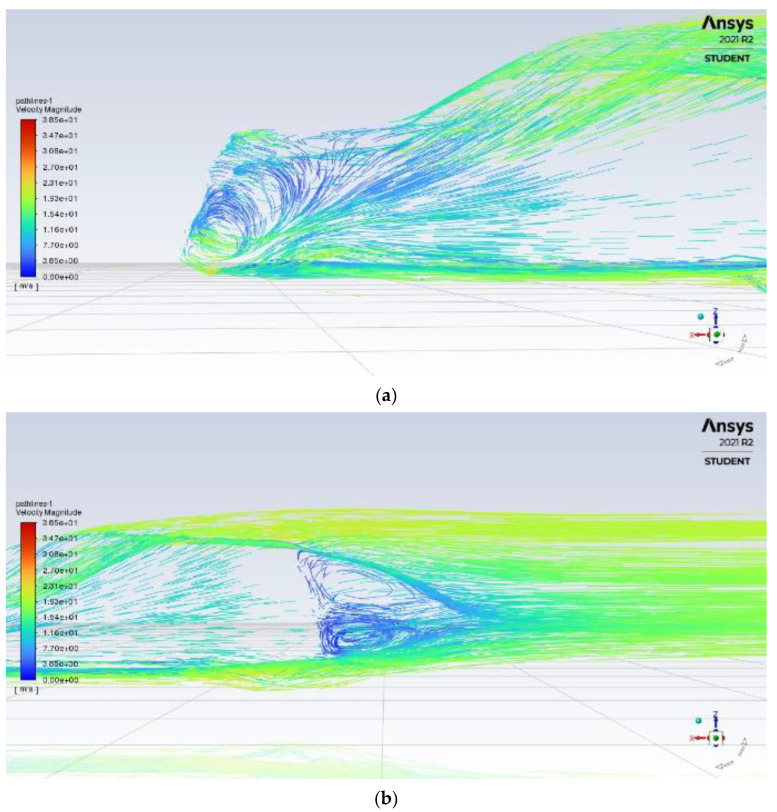
Velocity magnitude of the adjusted model at (**a**) front side, (**b**) rear edge of spoiler.

As the individual modifications have been tested, the adjusted model with all modifications attached is analysed. The structure of the adjusted model is shown in Figure 13. The impact of the fins and spoiler can be seen in Figure 14, with flow separation commencing at the edge of the spoiler and the creation of side vortices limited by the fins. As a result, the upper half of the circulation zone is greatly reduced, minimizing rear drag. As the air reaches the diffuser, it reaches a high-pressure area, slowing the airspeed; this transition creates a negative lift, pushing the car onto the ground and creating downforce. Air is pushed away from the rear of the car, to minimize drag and air flowing towards the circulation zone. The turbulence from the bottom of the car is reduced through the use of a diffuser and splitter allowing smoother air to flow from the bottom; similarly, turbulence from the top and sides is minimized due to the spoiler and fins. As a result, the circulation zone in the rear is smaller in size and less turbulent, contributing less drag to the car.

### 3.7. Aerodynamic Improvement Analyses

Aerodynamic results are shown in Table 3. After adding the front splitter, a 9% reduction in drag with a 73% reduction in lift can be observed. The bottom wake of model 2 is much smaller with less turbulence, which can reduce the size of the recirculation zone at the rear and improve drag. In model 3, from the aerodynamic results improvements in drag reductions are minimal; however, coefficient of lift is greatly reduced, and high values of lift can be dangerous, as air creates an upward force separating the car from the ground. Instead, improvement in downforce causes the air to push the car towards the ground, improving safety and stability at higher speeds and making the car easier to handle. In model 4, the spoiler’s objective in reducing air speed can be seen, although the car’s lift has increased. In model 5, the drag coefficient, drag force and lift coefficient decreased by 3.16%, 3.16% and 80.8%, respectively. Model 6 has improved all aerodynamic properties from the base model, whilst obtaining the lowest drag coefficient value and reducing lift. As the model produces negative lift (downforce) the car’s stability is improved at higher speeds, as vertical air pushes the car onto the ground providing more grip; however, downforce value must be monitored, as a value too large can provide too much force for the car to accelerate spoiling fuel consumption. The reduction in drag will reduce the air resistance towards the car, minimizing the amount of power needed to move the car and increasing its efficiency. Finally, Model 6 has obtained a 10% reduction in the drag coefficient.

### 3.8. Discussion

According to the achieved results, the proposed adjustments reduce the overall drag at the surface. According to Nixon’s study [31], the drag force is proportional to entropy generation. The author used an extended form of Crocco’s equation, which relate aerodynamic loads to entropy production. Consequently, by solving Crocco’s equation at the airfoil surface, the entropy production can be estimated as a function of additive terms. These terms include the effects of vorticity flux, viscosity and energy losses on entropy production. Based on their studies, we can conclude that the common factor between these terms is the Mach number, which is proportionally related to the entropy production. For our study, the performed enhancements reduced the stream velocity. As a result, the Mach number and the total entropy production were reduced. In addition, the entropy production increases with the tangential part of the drag force, which decreases with the stream velocity reduction for the electric vehicles in our studies.

## 4. Conclusions

The development of electric vehicles plays a significant role in carbon mitigation in transport sectors worldwide. Due to the limit of battery technologies, it is essential to understand the aerodynamic performance of electric vehicles. The present study developed an advanced computational fluid dynamics modelling to optimize the design of an electric hatchback and improve the range of electric vehicles. The experimental data were used to validate the numerical modelling. The simulation predicted the experimental result within 4.36% error, representing a reasonable agreement. The study has highlighted various modifications in reducing drag coefficient, compared to the base design of the electric hatchback. The most relevant findings of the research can be stated as follows:

- The optimized design by adding the front splitter, rear diffuser, rear spoiler and rear fins decreases the hatchback’s drag coefficient and improves safety and stability at higher speeds, making the car easier to handle.
- The optimization contributed to a 10% reduction in the drag coefficient of the electric hatchback and improved the aerodynamic performance and the range of electric vehicles.
- The second model with front spitter has an insignificant improvement limited to 1.17%, compared with 11.18% when the rear diffuser was involved separately. In addition, the lift coefficient was significantly reduced to 73%, providing better stabilities and accounting for the safety measurements, especially at high velocity.

## Figures and Tables

**Table 1:** Model dimensions for the CFD modelling.

Car Dimensions	Value (m)
Length	4.88
Width	1.85
Height	1.5

**Table 2:** Comparison of the drag force coefficient between experimental and numerical results.

Experimental Value, *C_D_*	Simulation, *C_D_*	Error (%)
0.298	0.311	4.36%

**Table 3:** Aerodynamic improvements on models.

Model#	Model Modifications	Drag Coefficient	Drag Force	Lift Coefficient
-	-	-	(N)	-
1	base model	0.4642	381.5	0.003681
2	front splitter	0.4224	347.1	0.0009733
3	rear diffuser	0.4640	381.3	−0.003848
4	rear spoiler	0.4501	369.9	0.003911
5	rear fins	0.4496	369.4	0.0007050
6	overall	0.4175	343.0	−0.003455

## Data Availability

The research data supporting this publication are provided within this paper.

## Nomenclature

A	Surface area [m^2^]	v	fluid velocity in y direction [m/s]
C_1_, C_2_, C_1__ε_, C_3ε_	Model constant	w	fluid velocity in z direction [m/s]
C_D_	Drag coefficient	Y_M_	fluctuating dilatation [J]
C_L_	Lift coefficient	*Greek symbols*	
G_b_	Buoyancy turbulence kinetic energy [J]	ε	turbulent dissipation rate
G_k_	Mean velocity gradients turbulence kinetic energy [J]	ν	kinematic viscosity [m^2^/s]
k	Turbulent kinetic energy [J]	ρ	density [kg/m^3^]
p	Pressure [Pa]	σ_k_, σ_ε_	Model constant
S,S_Mx_,S_My_,S_Mz_S_k_,S_ε_	Source items	μ	dynamic viscosity [Pa·s]
t	time [s]	*Subscripts*	
u	fluid velocity in x direction [m/s]	ICE	Internal Combustion Engine
